# Impact of temperature-dependent phage expression on *Pseudomonas aeruginosa* biofilm formation

**DOI:** 10.1038/s41522-021-00194-8

**Published:** 2021-03-16

**Authors:** Karishma Bisht, Jessica L. Moore, Richard M. Caprioli, Eric P. Skaar, Catherine A. Wakeman

**Affiliations:** 1 Department of Biological Sciences, Texas Tech University, Lubbock, TX USA; 2 Department of Chemistry, Vanderbilt University, Nashville, TN USA; 3 Department of Pathology, Microbiology, and Immunology, Vanderbilt University Medical Center, Nashville, TN USA

**Keywords:** Biofilms, Microbial ecology, Pathogens

## Abstract

*Pseudomonas aeruginosa* is a ubiquitous opportunistic pathogen that forms robust biofilms in the different niches it occupies. Numerous physiological adaptations are required as this organism shifts from soil or aquatic environments to a host-associated lifestyle. While many conditions differ between these niches, temperature shifts are a factor that can contribute to physiological stress during this transition. To understand how temperature impacts biofilm formation in this pathogen, we used proteomic and transcriptomic tools to elucidate physiological responses in environment-relevant vs. host-relevant temperatures. These studies uncovered differential expression of various proteins including a phage protein that is associated with the EPS matrix in *P. aeruginosa*. This filamentous phage was induced at host temperatures and was required for full biofilm-forming capacity specifically at human body temperature. These data highlight the importance of temperature shift in biofilm formation and suggest bacteriophage proteins could be a possible therapeutic target in biofilm-associated infections.

## Introduction

Bacteria form highly adaptable multicellular communities called biofilms^1^. A biofilm is a group of microorganisms that adheres to a surface by utilizing extracellular polymeric substances (EPS). The ability of bacteria to form a biofilm varies depending on their environment and bacterial taxonomy^2,3^. Studies have shown that exposing biofilms to different environmental factors can induce the expression of different sets of genes and ultimately yield distinct biofilm morphologies^4^. Biofilms can be found naturally attached to rocks within streams or rivers where they can beneficially contribute to the ecosystem^5^. However, biofilms are also found growing within industrial settings, such as water pipes, that can cause operational problems and negatively impact industry^6^. Similarly, the biofilms naturally associated with the human body can range from beneficial to pathogenic^5,7,8^.

*Pseudomonas aeruginosa* is a ubiquitous pathogen associated with hospital-acquired infections. It has been classified by the Centers for Disease Control as a serious threat due to its high level of antibiotic resistance, especially when growing in its clinically relevant biofilm form^5,9^. Its ability to form vigorous biofilms can contribute to both antibiotic resistance as well as resistance to the human immune system^10^. In general, biofilms account for almost 80% of chronic microbial infections in the human body, and biofilm-forming microbial cells are up to 1000 times more antibiotic resistant than the planktonic counterparts^8^. The EPS matrix surrounding the microbial community consists of proteins, lipids, polysaccharides, and extracellular DNA and helps the bacteria to survive under harsh conditions^10^.

The impact of temperature on bacterial pathogens is well-established. For example, the elevated host temperature triggers the production of *Yersinia* species virulence factors^11^. This phenomenon has also been noticed in bacterial pathogens for plants where virulence genes are repressed at elevated temperatures^12^. These findings highlight the importance of temperature-based regulation of gene expression on host colonization and disease progression with a reverse effect in the case of plant pathogens and human pathogens. Other studies have shown an association between temperature and biofilm formation, regulated by cyclic di-GMP signaling in opportunistic bacterial pathogens, such as *Burkholderia pseudomallei* and *P. aeruginosa*^13,14^. Due to the wide range of temperatures experienced by *P. aeruginosa* as it transitions from the aquatic or soil environment into the human host, previous studies have already begun to explore thermal regulation in this microbe^4,14,15^. One particularly thorough study explored the transcriptomic response in planktonic cells shifting between 22 and 37 °C^4^. Other studies focusing on the impact of temperature shifts on biofilm cells only used short-term exposure to non-physiological heat stresses as a form of biofilm mitigation^15^. Another recent study demonstrated that thermoregulation of biofilm formation in *P. aeruginosa* was strain dependent but consistently more robust at lower temperatures^14^. Our research seeks to expand on these interesting studies by identifying biofilm-specific adaptations of *P. aeruginosa* at both external environmental temperatures and host-relevant temperatures since the biofilm lifestyle of this organism has been intricately linked to its ability to colonize both of these niches.

Biofilm formation in *P. aeruginosa* depends on various factors and the genes can be differentially regulated in the presence of varied environmental conditions^16,17^. Both genetic background and environmental factors can have an effect on the transcriptional profiles and evolutionary trajectory of this pathogen^18^.Temperature shifts can be detected by bacteria utilizing various sensors involving temperature-driven conformational changes in either DNA, RNA, or proteins^19–21^. For example, RNA thermometers have been reported to be involved in thermoregulation in *P. aeruginosa*^22^. The stress conditions associated with the temperature shifts that *P. aeruginosa* experiences as it transitions from the external environment into the host could contribute to differential protein expression that impacts biofilm formation. By understanding how *P. aeruginosa* regulates these proteins in response to both environmental and host-relevant temperatures, we can potentially identify novel therapeutic strategies to combat the biofilm-strengthening proteins with specific relevance to either the host or the industrial environment.

One exciting area of exploration for therapeutic potential is bacteriophages, as these bacteria-targeting viruses can eradicate recalcitrant microbial populations, such as those growing within biofilms and/or associated with certain infections^23,24^. Conversely, some bacteriophages have recently been found to contribute to the structural integrity of biofilm via incorporation into the EPS matrix, which aids in biofilm adherence to surfaces and tolerance to stress conditions^24^. Pf bacteriophage in particular has been associated with influencing *Pseudomonas* phenotypes by not only having an effect on biofilm formation and antibiotic resistance but also impacting mammalian immune responses during infection^25,26^. Differential phage reactivation has been associated with stress responses linked to temperature shifts in other microbes^27–29^. Similarly, induction and increased release of Pf phages has been associated with stress conditions experienced by the bacterial host^25^. In addition, the bacterial antiviral CRISPR immune system in *P. aeruginosa* is impacted by temperature^30^. Therefore, it is possible that differential phage reactivation or induction could occur within *P. aeruginosa* which could influence biofilm formation in this microorganism. *P. aeruginosa* is a problem for both human health^31^ (in the case of chronic infections) and industry^6^ (such as the pipe-associated biofilms). Exploring the role of temperature fluctuations and temperature-responsive phage proteins on the development of *P. aeruginosa* biofilms can be beneficial as fluctuations in temperature are a major component of its transition from an environmental lifestyle to a host-associated lifestyle.

## Results

### Temperature impacts biofilm architecture

**Fig. 1:**
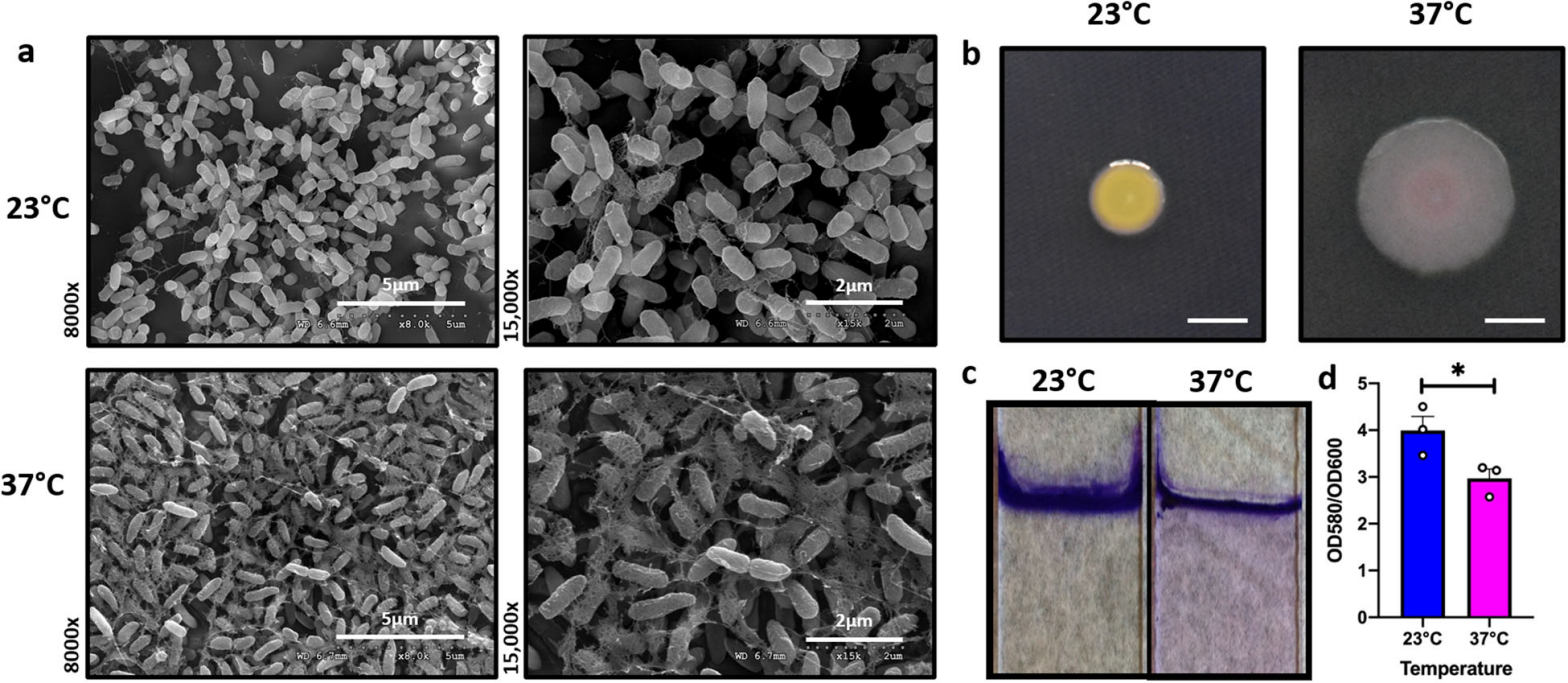
Growth temperature influences biofilm architecture in *P. aeruginosa*. **a** Comparison of the scanning electron microscopy images of *Pseudomonas aeruginosa* wild-type biofilms grown for 48 h at 23 and 37 °C. Images show ×8000 and ×15,000 magnification and are representative of three independent experiments. **b** Congo red binding assay. Extracellular matrix production by the wild type was evaluated on tryptone agar plates containing Congo Red and Coomassie brilliant blue G after incubation at 23 and 37 °C for 72 h. Representative images of the colony morphologies of WT PA14 are shown. Scale bar: 5 mm. **c** Crystal violet staining revealed subtle temperature-dependent architectural differences. **d** The biomass of the biofilm was higher at 23 °C when compared to 37 °C. Bars represent the mean of three biological replicates performed on different days. The mean of each biological replicate was based on three technical replicates. Error bars represent the standard error of mean of the biological replicates. Unpaired *t*-test (two-tailed) was used to measure statistical significance. *p = 0.0146.

Biofilms acquire unique architecture (such as the re-arrangement of the EPS matrix and the cells within) to persist in a wide range of environments, including the host environment^32^. Because the growth temperature is a striking difference between the external/natural environment of *P. aeruginosa* vs. the conditions during a human infection, we wanted to determine the effect of temperature on biofilm architecture of *P. aeruginosa* strain UCBPP-PA14 (PA14) at 23 and 37 °C using scanning electron microscopy (SEM), where 23 °C represents a temperature experienced by *P. aeruginosa* in environmental niches and 37 °C mimics the host body temperature. These studies revealed a striking difference in EM-visible matrix production at both these temperatures. At 23 °C, the matrix appeared to be scattered while at 37 °C, it was denser with cells entangled within the matrix (Fig. 1a). We further studied the architecture of biofilms grown at different temperatures using confocal microscopy. The overall architecture of 37 °C appeared slightly more complex while the 23 °C biofilms were slightly flatter (Supplementary Fig. 1). These subtle architectural differences may be due to the EPS differences in these conditions as observed by SEM.

This potential difference in the EPS matrix production by PA14 at both temperatures was further evaluated using the Congo red binding assay. This assay has previously been used to study the morphology of *P. aeruginosa*^33^. Tryptone agar plates containing Congo Red and Coomassie brilliant blue G were used for this experiment and were incubated at 23 and 37 °C for 72 h (Fig. 1b). The PA14 colonies looked morphologically different at both temperatures, with less dye absorption at lower temperature compared to 37 °C. At the host temperature, the colony was more bluish-reddish, indicative of changes in EPS composition since these dyes are known to associate with different EPS components^33^. We also studied the biofilm robustness of *P. aeruginosa* at these two temperatures using the established crystal violet (CV) staining method^34^. The CV staining revealed subtle temperature-dependent architectural differences (Fig. 1c). For example, the biomass at the air-liquid interface appeared thicker in the biofilm formed at 23 °C but the biofilm formed at 37 °C displayed visible but diffuse surface-adhered growth extending down the length of the submerged glass surface. On quantifying the biofilm formed at each temperature, we found an increase in biofilm formation at 23 °C when compared to 37 °C (Fig. 1d), which is consistent with findings by other groups that this environmental microbe and opportunistic pathogen forms slightly more dense biofilms at lower temperatures^14^. However, upon quantifying biofilm-associated colony-forming units (CFUs), we determined that biofilms grown at either temperature have comparable viable cell numbers (Supplementary Fig. 2a).

### Temperature influences protein expression in biofilm

**Fig. 2:**
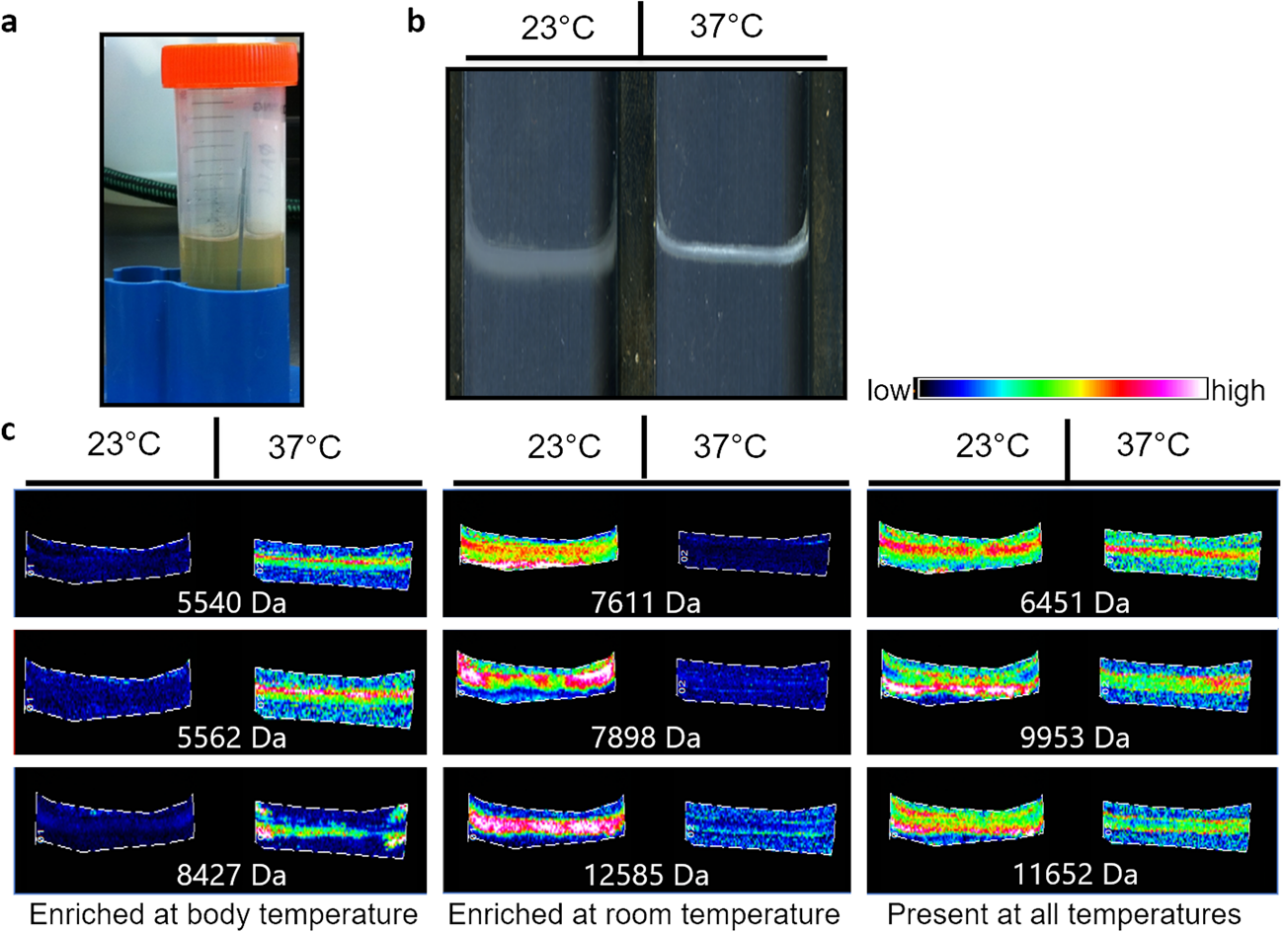
Proteomic analysis reveals global changes in biofilm-associated protein driven by differences in growth temperature. **a** Pellicle biofilms were grown on glass slides at different temperatures. **b** Biofilms were grown on conductive glass slides for analysis by matrix-assisted laser desorption/ionization imaging mass spectrometry (MALDI IMS). **c** MALDI IMS revealed dramatic temperature-dependent changes in protein abundance and distribution in pellicle biofilms.

To investigate the presence of the different proteins expressed at environmental vs. host temperature we used matrix-assisted laser desorption/ionization imaging mass spectrometry (MALDI IMS)^35^. This technology has been extensively used in the biofilm field to visualize the heterogeneity of proteins existing within the microbial communities^36^. It has previously been used to detect the presence of different proteins in the distinct regions of the biofilm formed by *P. aeruginosa*^37^. Pellicle biofilms (biofilms that form at the air-liquid interface) were grown on conductive glass slides for analysis at different temperatures by MALDI IMS (Fig. 2a, b). We saw that the biofilms grown under different temperature conditions exhibit dramatically different protein expression profiles. Some proteins were enriched at host temperature (37 °C), others were enriched at environmental temperature (23 °C), and many proteins were found to be unchanged between these two conditions (Fig. 2c and Supplementary Fig. 3). Overall, these data demonstrate a dramatic shift in biofilm-associated protein production at these two temperatures. As various proteins have been shown to be integral components of EPS in numerous microbes, these findings could be indicative of temperature-specific EPS adaptations.

### Phage Pf1 expression is induced in biofilms at host temperature

**Fig. 3:**
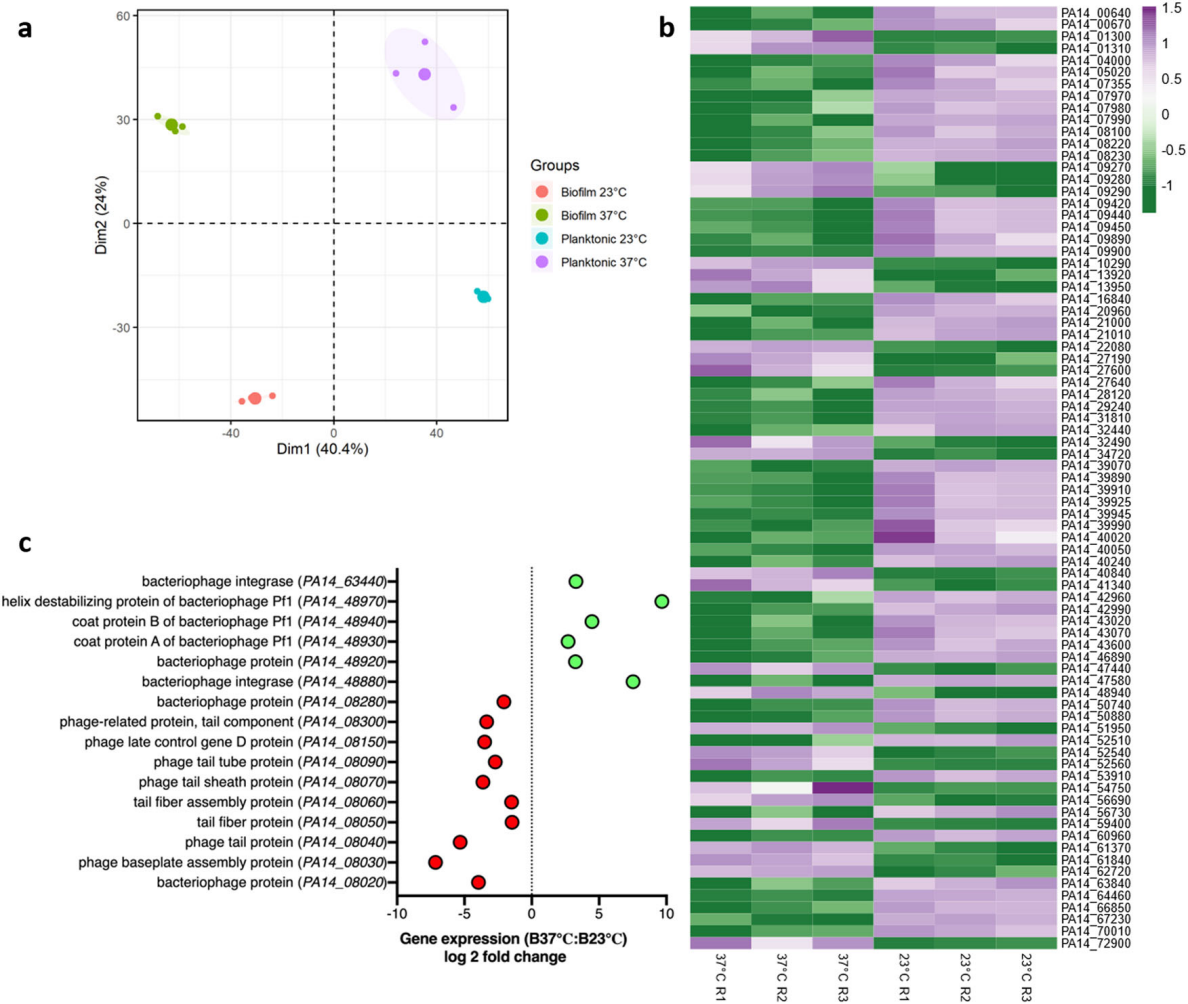
Growth temperature as well as biofilm and planktonic growth modes drive significant shifts in gene expression of various gene categories including phage operons. RNA extraction was performed for *P. aeruginosa* cells grown at 23 and 37 °C (in triplicates). The reads were then analyzed using Rockhopper software followed by calculating the fold change for the phage proteins at each temperature. **a** Transcriptomic multivariate analyses for *P. aeruginosa* PA14 grown in biofilm and planktonic form at 23 and 37 °C. Principal component analysis (PCA) plot is shown. **b** Heat map showing genes regulated by temperature in biofilm state only. Each row in the heat map corresponds to differential expression of the gene at the two temperatures, whereas each column corresponds to one replicate (a total of three) grown at the two temperatures in the biofilm state. Gradient scale is representing expression levels which were preprocessed before plotting by natural log transformation with purple showing highest expression and green with lowest expression. **c** Transcriptomic data analysis shows that some of the phage proteins are expressed more at 37 °C (green) while others are expressed higher at 23 °C (red).

While the MALDI IMS findings supported that the overall biofilm proteome of *P. aeruginosa* is highly impacted by temperature shifts, we sought to determine some of the specific gene expression changes that might contribute to temperature-driven differences in EPS composition. Therefore, we performed transcriptomic analyses of both planktonic and biofilm-associated cells at 23 and 37 °C. Principal component analysis for *P. aeruginosa* PA14 grown in biofilm and planktonic form at 23 and 37 °C revealed a notable clustering effect of the replicates for biofilm as well as the planktonic stage (Fig. 3a). Genes were differentially expressed at the two temperatures in both the planktonic and biofilm state. A total of 175 and 189 genes were differentially regulated in planktonic and biofilm states, respectively, at the two temperatures (Supplementary Fig. 4). At 23 °C, a total of 464 genes were differentially regulated in biofilm vs. planktonic growth state and at 37 °C, a total of 373 genes were differentially regulated in biofilm vs. planktonic growth state (Supplementary Fig. 4). We used the *P. aeruginosa* Community Annotation Project (PseudoCAP) function class assignments (http://pseudomonas.com/pseudocap) to categorize the differentially expressed genes into different functional classes^38^. 94 genes were upregulated in the planktonic state, at environment-relevant temperature while 81 genes were downregulated for this state. In the case of biofilm, 91 genes were upregulated while 98 were downregulated at 23 °C vs. 37 °C (Supplementary Fig. 5a, b). On comparing the gene expression at the same temperature between biofilm and planktonic transcripts, we found a higher number of genes differentially expressed because of this lifestyle switch. At 23 °C, we identified 172 genes upregulated and 292 genes downregulated in biofilm vs. planktonic cells. However, at 37 °C, there were 199 genes upregulated and 176 genes downregulated between biofilm and planktonic cells (Supplementary Fig. 6a, b). We were also able to find genes that were regulated by temperature exclusively in the biofilm state (Fig. 3b) and exclusively in the planktonic state (Supplementary Data 1). There were also genes specifically differentially expressed in biofilm vs. planktonic at the environmental temperature only (Supplementary Data 2) and genes differentially expressed in biofilm vs. planktonic at host temperature only (Supplementary Data 3). Interestingly, there were 35 genes that were expressed solely dependent on temperature fluctuation and 148 genes which were solely dependent on the planktonic vs. biofilm switch (Supplementary Data 4 and 5). All the raw count data associated with differential expression of genes due to thermoregulation in *P. aeruginosa* is also made available (Supplementary Data 6).

When quantifying CFUs in biofilms grown at 37 and 23 °C, we encountered small colony variants (SCVs) specifically in *P. aeruginosa* biofilms grown at 37 °C (Supplementary Fig. 2b, c). The SCVs have previously been reported to be present in the lung adapted *P. aeruginosa* strains exhibiting slow-growing properties, enhanced biofilm formation, and more exopolysaccharide production^39^. In addition, Pf bacteriophage has been previously linked with SCV formation in *P. aeruginosa*, a phenotype promoting persistence at host temperatures^25,40^. The known association between SCVs and Pf phage directed our focus to some of the phage genes found to be differentially regulated in our transcriptomics data sets. Interestingly certain phages were found to be upregulated at 37 °C while others were expressed higher at 23 °C (Fig. 3c). For example, a classic tailed bacteriophage operon encompassing genes ranging from PA14_08020 to PA14_08280 was more highly expressed at environmentally relevant temperatures (23 °C) whereas the filamentous Pf1 phage proteins ranging from PA14_48880 to PA14_48970 were expressed higher at host-relevant temperatures (37 °C). Of particular interest was PA14_48940, the Pf1 bacteriophage coat B protein (*coaB*), which was the only phage protein that was found to be expressed higher, exclusively in the biofilm state, at the host temperature vs. environmental temperature (Fig. 3c). This protein has been previously reported to be participating in maintaining the structural integrity of the biofilm matrix^24^. Since our transcriptomic analysis showed an increase in the expression of this phage protein at 37 °C, we hypothesized that it could be contributing to the temperature-specific EPS adaptation in *P. aeruginosa* biofilms.

### Phage protein is specifically required for biofilm formation at 37 °C and not 23 °C

**Fig. 4:**
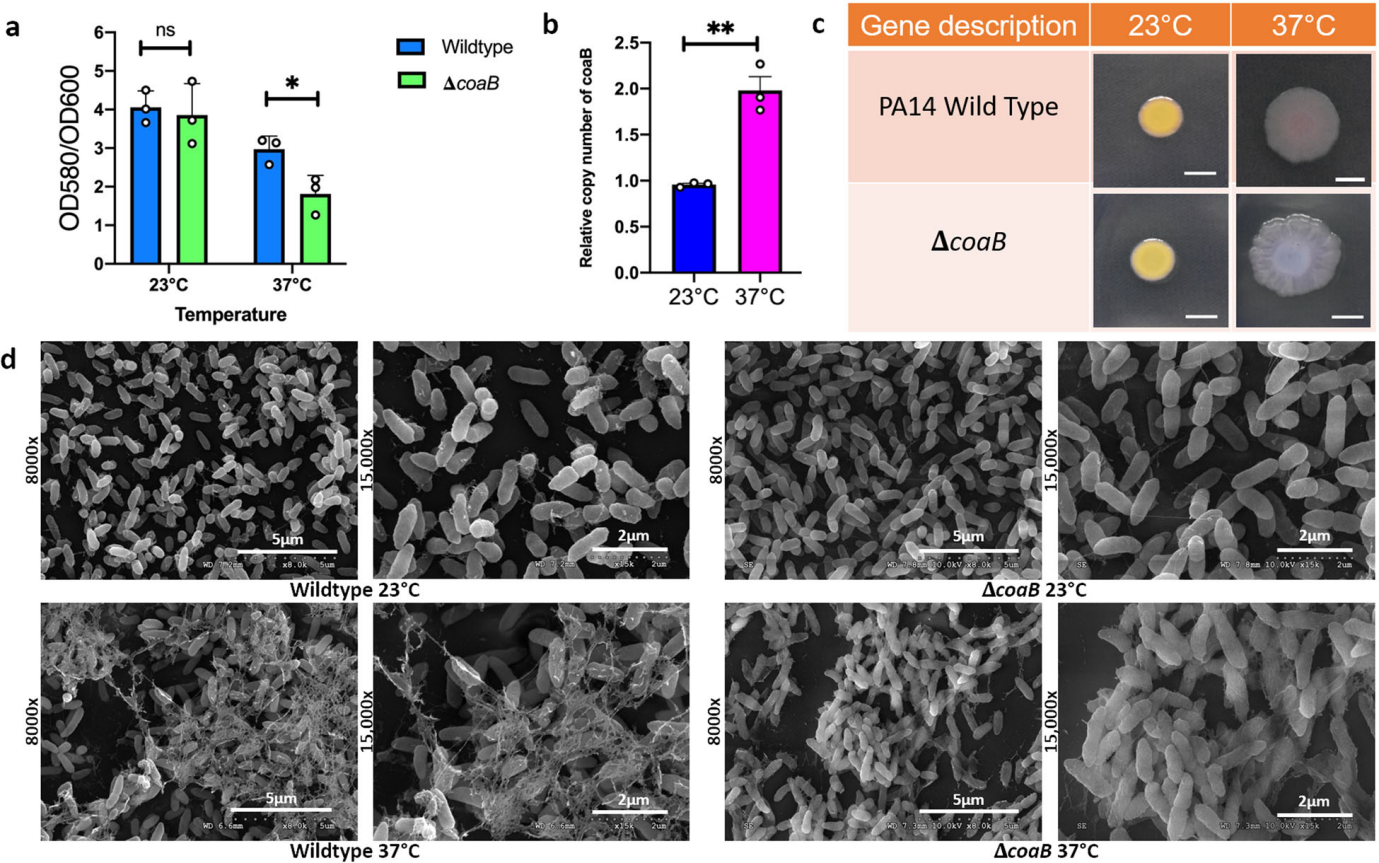
The Pf1 filamentous phage coat B protein specifically contributes to *P. aeruginosa* PA14 biofilm EPS at human body temperature but not at room temperature. **a** Crystal violet staining was performed to assess air-liquid biofilm formation in a 96-well microtiter plate at 48 h. Strains were grown in LB broth at appropriate temperatures. Bars represent the mean of three biological replicates performed on different days. The mean of each biological replicate was based on three technical replicates. Error bars represent the standard error of mean of the biological replicates. Unpaired *t*-test (two-tailed) was used to measure statistical significance. *p = 0.038, ns, *p* = 0.6071. **b** qPCR was performed on DNA extracted from biofilm grown for 48 h at 23 and 37 °C using primers specific for *coaB* and the housekeeping gene *rplU* using an established mechanical dissociation method^42^. Bars represent the mean of three biological replicates performed on different days. The mean of each biological replicate was based on three technical replicates. Error bars represent the standard error of mean of the biological replicates. Unpaired *t*-test (two-tailed) was used to measure statistical significance. **p = 0.0028. **c** Congo red binding assay. Extracellular matrix production by the wild type and mutant strain was evaluated on tryptone agar plates containing Congo Red and Coomassie brilliant blue G after incubation at 23 and 37 °C for 72 h. Representative images of the colony morphologies of WT PA14 and the **Δ***coaB* mutant are shown. Scale bar: 5 mm. **d** Comparison of the scanning electron microscopy images of *Pseudomonas aeruginosa* wild-type biofilms and **Δ***coaB* mutant biofilms grown for 48 h at 23 and 37 °C. Images show ×8000 and ×15,000 magnification and are representative of three independent experiments.

Filamentous phages are abundantly present at sites of chronic infection along with *P*. *aeruginosa* and CoaB is a major coat protein of this phage^24,25^. Since our transcriptomic data pointed towards the presence of this phage protein at host temperature, we were particularly interested to investigate the role of CoaB protein of bacteriophage Pf1 in biofilm formation. For our experiments, we used the *coaB* mutant derived from a commercially available transposon mutant library of PA14^41^. The identity of the mutant was confirmed by arbitrary PCR followed by sequencing. We performed the established CV staining protocol to study the biofilm formed by this mutant at the two temperatures. *coaB* mutant strain showed a decrease in biofilm formation relative to the wild type at 37 °C, suggesting a role of this protein in biofilm formation at this particular temperature (Fig. 4a). However, no such difference was observed in the biofilm formation at 23 °C, indicating that this protein is dispensable for robust biofilm formation at lower temperatures. This decrease in overall biofilm biomass was accompanied by a decrease in biofilm-associated CFUs in the *coaB* mutant specifically at 37 °C with no such decrease observed for overall planktonic growth of this mutant in any of the conditions tested (Supplementary Fig. 7a–c). Interestingly, we still observed the induction of the SCV phenotype in the *coaB* mutant at 37 °C (Supplementary Fig. 7b). This indicates that even if the phage induction and SCV formation are linked as previously reported^40^, the SCV phenotype is not dependent on the presence of intact Pf phage secretion in the case of temperature-induced SCV formation. We also quantified the abundance of the Pf1 phage DNA genome relative to the PA14 genome in the biofilm formed by *P. aeruginosa* at both 23 and 37 °C following an established method for determining Pf1 production levels^42^. Similar to our transcriptomics results, the qPCR data also showed us a higher copy number of *coaB* gene in biofilm formed at 37 °C than the one formed at 23 °C (Fig. 4b).

We next wanted to examine the difference in the EPS architecture in this mutant when grown at the two temperatures. We first used the Congo red binding assay to evaluate the colony morphology of both the wild type and mutant at both the temperatures. The *coaB* mutant colony looked similar to the PA14 colony at 23 °C. However, at 37 °C *coaB* mutant colony looked morphologically different than the wild type with a more wrinkled colony appearance and slight differences in coloration on the Congo Red plates (Fig. 4c). Because the agar colony biofilm in a traditional Congo Red assay is a vastly different growth condition than the pellicle biofilms that were otherwise being analyzed in this study, we also quantified the congo red uptake by pellicle biofilm formed at 23 and 37 °C for both the wild type and mutant. There was no significant difference between the mutant and the wild type at 23 °C, while at 37 °C the *coaB* mutant had less uptake of the congo red dye when samples were normalized relative to the overall biomass (Supplementary Fig. 8). We next used SEM to see the difference in the biofilm formed by the mutant at both these temperatures. The matrix of the mutant cells looked smoother in comparison to the wild-type matrix which showed the presence of more filament-like structures at 37 °C. No such architectural differences were observed for the mutant matrix at 23 °C (Fig. 4d). Overall, these results show the importance of this phage protein at the host temperature with no impact on biofilm formation at the environmentally relevant temperature of 23 °C.

## Discussion

Biofilm-associated infections are particularly recalcitrant to clearance by both antimicrobial therapy and immune function^43,44^. In addition, the emergence and spread of antibiotic resistance have become a global threat and there is evidence that agricultural and industrial use of antibiotics is a contributor to the aggregation of resistance in the environment^45^. Therefore, studying biofilm adaptations specific to the human host or specific to industrial/environmental (i.e., soil/root associated or aquatic) biofilms will enable the development of antimicrobials targeted for specific use in each case. Such practices will help to prevent the unnecessary spread of microbial resistance to clinically relevant antibiotics. In this study, we wanted to investigate the effect of temperature on biofilm formation in *P. aeruginosa*, an opportunistic pathogen, which is known to form biofilm in various environments.

We hypothesized that biofilm grown at different temperatures will possess different structural adaptations and have distinct genetic requirements. Our SEM micrographs and CLSM images clearly show a distinct difference in the EPS matrix in the biofilm formed by *P. aeruginosa* at the environment and host-relevant conditions, supporting our hypothesis. The Congo red binding assay further bolstered our hypothesis by showing differences in EPS dye association with colony biofilms at these temperatures. MALDI IMS, a protein imaging technique that has been used previously to study the protein distribution in biofilms^37,46,47^, further demonstrated that pellicle biofilms grown under different temperature conditions exhibit different protein expression profiles, which also supported the possible presence of unique temperature-specific biofilm adaptations.

Next, we analyzed the transcriptomic data for both planktonic and biofilm-associated cells at both 23 and 37 °C. We found that the expression of several genes were uniquely impacted by only temperature fluctuation whereas others were unique to only planktonic to biofilm switch. Our observation of SCVs specifically in 37 °C biofilms directed our attention to certain phage genes in our transcriptomics data sets that had previously been associated with the SCV phenotype^24^. Indeed, our transcriptomic data supported a temperature-based regulation mechanism for phage proteins like coat b (CoaB) protein of Pf1 prophage, which participates in biofilm function and persistent infection within the host^24,25^. These data combined with our observation that the *coaB* mutant was specifically defective in biofilms formation at host-relevant temperatures adds to the growing collection of literature that demonstrates that Pf1 bacteriophage provide structural integrity to *P. aeruginosa* biofilms and indicates that this phenomenon may be specifically applicable to microbial physiology during infection. This finding may indicate different stressor susceptibilities at specific temperatures. For example, presence of the Pf phage in the host temperature biofilms may yield temperature-specific resistance to aminoglycoside as the incorporation of this phage in EPS has been associated with increased tolerance of this class of antibiotics^24^.Our findings are consistent with previous studies that have found high abundance of these phages at sites of chronic infection as well of the literature that demonstrates the contribution of Pf phages to *Pseudomonas* pathogenesis^24,48,49^. Pf phages can not only help this pathogen to tolerate antibiotic exposure but can also play an important role in host colonization and combatting mammalian immune system^25,26,50^. Other promising roles of Pf bacteriophage include inhibiting the growth of a fungal biofilm, providing structural integrity to *Pseudomonas* biofilm matrix and its role as an iron chelator in biological systems^51–53^, thus highlighting its synergistic relationship with *P. aeruginosa*. The ssDNA (single strand) genome of the Pf phage consists of several genes, of which two encode for coat proteins, CoaA (minor coat protein) and CoaB (major coat protein), and together these genes help this phage to replicate and integrate into the bacterial genome^25,54^. In our study, we found that CoaB, the major coat protein of Pf1 was not only important for biofilm formation but its expression was thermally regulated. Future studies on understanding how *P. aeruginosa* regulates its phage proteins in response to both external and host-relevant temperatures can potentially identify novel therapeutic strategies to exploit biofilm-targeting phages while combatting the effects of biofilm-strengthening phage proteins. Because a recent study highlighted the role of temperature in biofilm formation for different *P. aeruginosa* strains^14^, it will also be important to broaden our understanding of the impact of phage thermoregulation on the biofilm formation in other *P. aeruginosa* strains in the future.

In addition to studying the strain-to-strain variation in temperature-specific biofilm adaptations, the identification of temperature-specific EPS adaptations may be applicable to numerous other opportunistic pathogens capable of forming biofilms in the environment as well as in the human body. For example, similar MALDI IMS results demonstrating dramatic shifts in the biofilm-associated proteome were observed for *Acinetobacter baumannii*, another environmental microbe known to opportunistically colonize the human host in different infections sites^55^ (Supplementary Fig. 9). It will be important for future studies to define temperature-specific stressor susceptibilities driven by the different EPS composition produced in different growth environments. Such studies could reveal ideal therapeutic targets for use in the clinic as well as ideal targets to enable the eradication of problematic biofilms in industrial settings.

## Methods

### Bacterial strains, media, and growth conditions

*P. aeruginosa* strain UCBPP-PA14, a highly virulent strain of *P. aeruginosa* originally isolated from a wound infection, was used in all experiments unless otherwise stated^56^. An isogenic mutant of PA14, the PA14/MrT7:: *PA14_48940* strain, carrying the Mariner transposon *MAR2Tx7* inserted at nucleotide 226 in *coaB* (PA14::*coaB*)^41^, was used to examine the importance of the bacteriophage coat protein B in biofilm formation. Strains were routinely grown overnight and maintained at 37 °C in Luria-Bertani (LB) broth. Gentamicin was added at 15 μg/mL to maintain the transposon in the PA14::*coaB* mutant. To calculate colony-forming unit for the biofilm cells, bacterial cultures were grown for 48 h at 23 and 37 °C in test tubes under static conditions. The planktonic cells were first aspirated out, followed by the addition of 1X PBS and sonication using a Fisher Scientific FB120 Sonic Dismembrator with CL-18 Probe, with a pulse of 20% amplitude for 30 s. Five microliters from the tube was then plated onto Luria Agar plates and left for overnight growth.

### MALDI IMS

20 μL of the overnight grown culture of PA14 was added into a fresh 20 mL LB broth, in a 50 mL falcon containing a glass slide. Biofilms were grown on indium-tin oxide-coated glass slides (Delta Technologies, Loveland, CO) for 48 h as described previously^57^. Matrix was applied to sample sections using a TM-Sprayer (HTX Imaging, Carrboro, NC). The matrix was sprayed onto the sections at a flow rate of 0.2 ml min^−1^ using a pushing solvent of 90% acetonitrile. The TM-Sprayer was operated at a speed of 1200 mm min^−1^ and at a nozzle temperature of 80 °C. The spray pattern was set to 2 mm spacing and eight passes of matrix were applied. Imaging mass spectrometry was performed in linear positive ion mode using an AutofleX Speed mass spectrometer (Bruker Daltonics, Billerica, MA) at a 200 μm spatial resolution. Fifty laser shots were acquired per pixel in random-walk mode in ten shot steps. Data were processed using fleXimaging version 4.1.

### Microtiter biofilm formation assay

To quantify and study biofilm formation at each temperature we used the previously established microtiter biofilm formation assay^34^. Wild-type *P. aeruginosa* PA14 and the mutant strain were grown overnight in a 96-well round bottom plate in 150 microliters LB broth with shaking at 220 rpm and 37 °C overnight. Next day, 5 μL of the overnight grown culture was transferred to a fresh 96-well plate with 145 μL of LB media. This was done for three replicates at three different days. The plates were incubated for 48 h at 23 and 37 °C. An absorbance at 600 nm wavelength was taken after 48 h using Synergy Hi5 Microplate Reader, Biotek. Planktonic cells were then aspirated out and the remaining biofilm was washed three times with 300 μL of PBS. This step helps remove unattached cells and media components that can be stained in the next step and significantly lowers background staining. Next, 200 μL of 100% ethanol was added to the wells and incubated for 15 min. The ethanol was then aspirated out completely and the plates are flipped upside down and left for drying. After the ethanol dried, 200 μL of a 1% solution of CV was added to each well of the microtiter plate. The microtiter plate was then incubated for 15 min at room temperature followed by rinsing it 3–4 times with water by submerging the plate in a tub of water, and blot vigorously on a stack of paper towels to get rid of all the excess water. The microtiter plate was then left to dry for 1–2 h. Finally, 150 μL of 30% acetic acid solution was added to each well of the microtiter plate to solubilize the CV. After an hour of incubation at room temperature, absorbance was taken at 580 nm. Using the biomass baseline, this reading was quantified and analyzed to produce readable data.

### Bacterial RNA extraction

20 μL of the overnight grown culture of PA14 was added into a fresh 20 mL LB broth, in a 50 mL falcon along with a microscope glass slide. This was left at static conditions at both 23 and 37 °C for 48 h. Three biological replicates were processed for the WT at each temperature. Next, the biofilm from the glass slides was scraped off using a tip and 700 μL of Qiazol lysis reagent. For disrupting or lysing bacterial cells, the mixture was added onto tubes with beads (Tough Microorganism Lysing Mix (RNase + DNase free, size—2 mL × 0.5 mm Verre)) and lysed in a VWR Bead Mill Homogenizer at 6500 rpm for 1 min (two rounds). For planktonic cells, the 48 h grown culture was centrifuged at 5000 rpm for 10 min, followed by discarding the supernatant and resuspending the pellet in 700 μL of Qiazol lysis reagent. RNA was then extracted using the RNeasy Minikit (Qiagen) according to the manufacturer’s recommendations, and the RNA solution was digested with the RNase-free DNase set (Qiagen), followed by on-column DNase digestion to eliminate any remaining traces of genomic DNA. The purified RNA was quantified using a Take3 plate reader (Synergy H1 microplate reader, Biotek). RNA samples with 1.8–2.2 ratio of absorbance at 260/280 nm were kept for further analysis. The samples were then sent to Genewiz for library prep and Illumina HiSeq. Only samples with an RNA integration number greater than 8.0 were used for cDNA library preparation.

### Analysis of the RNA-seq data

RNA-seq data were analyzed using Rockhopper software implementing reference-based transcript assembly with UCBPP-PA14 as a reference genome followed by calculating the fold change for the transcripts at each temperature^58^. The full accession number of the UCBPP-PA14 annotation that was used is NC_008463. Each data set was normalized by upper quartile normalization, and then transcript abundance was quantified using reads assigned per the kilobase of target per million mapped reads normalization method. The selection criteria for differential expression required genes to have a fold change of ≥2 and a *q* value of ≤0.05 to be considered significant. The *q* value was obtained by adjusting the *p* value using the Benjamini–Hochberg procedure^59^.

### Congo red binding assay

To phenotypically assess the different components of the EPS matrix, a colony morphology assay was performed as described previously^33^. Extracellular matrix production by *P. aeruginosa* PA14 and *PA14::coaB* was evaluated on tryptone agar plates containing Congo Red and Coomassie brilliant blue G after incubation at 23 and 37 °C for 72 h. Five microliters of overnight precultures was spotted on Petri plates containing 20 mL of the assay medium (1% tryptone, Congo red dye (40 µg/ml), Coomassie brilliant blue G dye (20 µg/ml) and 1% agar). Colonies were grown at 23 and 37 °C for 72 h. Images of the colonies were taken daily using the Nikon camera. To quantify Congo Red binding to pellicle biofilm, the assay was adapted from previously described protocols^60^. Briefly, 20 μL of the overnight grown culture of PA14 was added into a fresh 20 mL LB broth, in a 50 mL falcon along with a microscope glass slide and was left at 23 and 37 °C for 48 h. The biofilm was then scraped off the glass slide and resuspended in 1 mL of PBS. OD 600 was taken for all the samples. Forty micrograms per mililiters of Congo red dye was then added to each tube and incubated at 37 °C for 1 h. Samples were then centrifuged at 16,873 × *g* for 2 min, and supernatants were transferred to a clear 96-well plate for measurement of absorbance at 490 nm using a plate reader (Synergy H1 Microplate Reader, Biotek). PBS + 40 µg/mL Congo red was used as a control. Finally, the exopolysaccharide-bound Congo-red was quantified by subtracting the A490 value of the sample from the A490 value of the control and normalizing the final value to OD 600 reading. All experiments are carried out on three independent days using three replicates each.

### Biofilm imaging

Biofilms grown on glass slides for 48 h were imaged by confocal laser scanning microscopy (CLSM) (Olympus; FV3000). 20 μL of the overnight grown culture of PA14 was added into a fresh 20 mL LB broth, in a 50 mL falcon along with a microscope glass slide and grown for 48 h. The biofilm was then washed with PBS followed by staining with FilmTracer LIVE/DEAD Biofilm Viability kit (ThermoFisher). Next, biofilm was incubated with SYTO™ 9 and propidium iodide stains for 20 min in the dark at room temperature as per the manufacturer’s protocol. After that, the biofilm was washed with PBS and the slides were imaged using a OlympusFV3000 microscope with an objective lens of ×60 (oil). Each experiment included three independent biological replicates and three images were taken for each replicate. For each glass slide, five image stacks were taken with a z-step size of 0.7 μm. ImageJ software^61^ was used to calculate the number of live (SYTO9; green) and dead (propidium iodide; red) cells.

### Electron microscopy

For SEM, static cultures were grown in 50-mL conical tubes with circular glass coverslips semi-submerged in 2 mL of LB broth. Coverslip samples were handled and processed as described previously^62^. Upon removal of the culture medium, the coverslips were immediately flooded with 2·5% glutaraldehyde in 0·05 M sodium cacodylate and incubated at room temperature for 1 h. The fixative was removed and replaced immediately with 0.05 M sodium cacodylate to prevent sample dehydration. The coverslips were then incubated in osmium tetroxide for 15 min followed by dehydration with increasing concentrations of ethanol, ranging from 25 to 100 %, and CO_2_ critical point drying. Samples were carbon-coated and visualized with a Hitachi S-4300 scanning electron microscope.

### DNA extraction and quantification of phage by qPCR

To quantify the phage present in PA14 biofilm we used a protocol that was designed to extract phage from sputum samples and modified it as per our requirement^42^. Briefly, 20 μL of the overnight grown culture of PA14 was added into a fresh 20 mL LB broth, in a 50 mL falcon along with a microscope glass slide and grown for 48 h. The slide was then washed with PBS and biofilm was then scraped off the glass slide and resuspended in 200 μL of PBS. The suspension was then added to a 2 mL tube filled ~1/5 by volume with 1 mm of ceramic beads. For the rest of the protocol QIAamp DNA Minikit (QIAGEN) was used. 20 μL of Proteinase K and 200 μL of buffer AL was added to the biofilm cells suspended in 200 μL of PBS. Next, a VWR Bead Mill Homogenizer was used for mechanical disruption, at 6500 rpm for 1 min (two rounds). The homogenized mixture was then used for DNA extraction as per the manufacturer’s protocol.

The Pf1 phage ssDNA genome as well as the dsDNA genome of *P. aeruginosa* in biofilm were quantified by qPCR. Two microliters of the DNA extracted from biofilm samples was used as a template in 10-μL qPCR reactions. 5 μL of iQ SYBR Green Supermix (BioRad) and 2 μM primers were used in the final reaction mixture. Cycling conditions were as follows: 95 °C for 2 min, (95 °C for 15 s, 60 °C for 20 s) × 40 cycles on a Biorad Real-Time PCR system. To quantify Pf1 phage, the primers *coaB*-F (AACGCATCGCCAAGTTCAGC) and *coaB*-R (ACGATATAGCCGCCGATGC) targeting *coaB* were used. For *P. aeruginosa* quantification, primers targeting the 50S ribosomal subunit gene *rplU*, *rplU*-F (CAAGGTCCGCATCATCAAGTT), and *rplU*-R (GGCCCTGACGCTTCATGT) were used. All samples were run in triplicate and qPCR was performed on three independent days. The Pf1 phage copy number represent the measured Pf1 phage with the *P. aeruginosa* copy number subtracted to account for the detection of prophage DNA contained in the genome of *P. aeruginosa*.

### Statistical analyses

Statistical analyses were performed using GraphPad Prism 8.0 (GraphPad Software, Inc., San Diego, CA). Unpaired *t*-test (two-tailed) was used to calculate the statistical significance.

### Reporting summary

Further information on research design is available in the Nature Research Reporting Summary linked to this article.

## Supplementary information

Supplementary Information

Supplementary Data 1

Supplementary Data 2

Supplementary Data 3

Supplementary Data 4

Supplementary Data 5

Supplementary Data 6

Reporting Summary

## Data Availability

The raw RNA-seq data sets generated during this study are available through NCBI’s BioProject database under accession number PRJNA664520. The raw data sets generated by MALDI IMS are available upon request. The authors declare that all other relevant data supporting the claims of the paper are available either in the main text or Supplementary files.
