# Supplementary material for: Impact of temperature-dependent phage expression on Pseudomonas aeruginosa biofilm formation
Source: NPJ Biofilms Microbiomes. 2021 Mar 16;7:22. doi: 10.1038/s41522-021-00194-8 (PMC7966754; doi:10.1038/s41522-021-00194-8)
Supplement: Supplementary file 1 — Supplementary Information [file 41522_2021_194_MOESM1_ESM.pdf]

Supplementary Information: Tables

Supplementary Data 1: Table of 66 genes regulated by temperature in planktonic state only.

Supplementary Data 2: Table of 242 genes specifically differentially expressed in biofilm versus planktonic state at room temperature only.

Supplementary Data 3: Table of 146 genes specifically differentially expressed in biofilm versus planktonic state at host temperature only.

Supplementary Data 4: Table of 35 genes differentially regulated by temperature shift only regardless of biofilm versus planktonic growth state.

Supplementary Data 5: Table of 148 genes differentially expressed in biofilm versus planktonic growth state regardless of temperature.

Supplementary Data 6: Raw data and count table for all PA14 genes in the RNA-seq analysis.

Supplementary Information: Figures

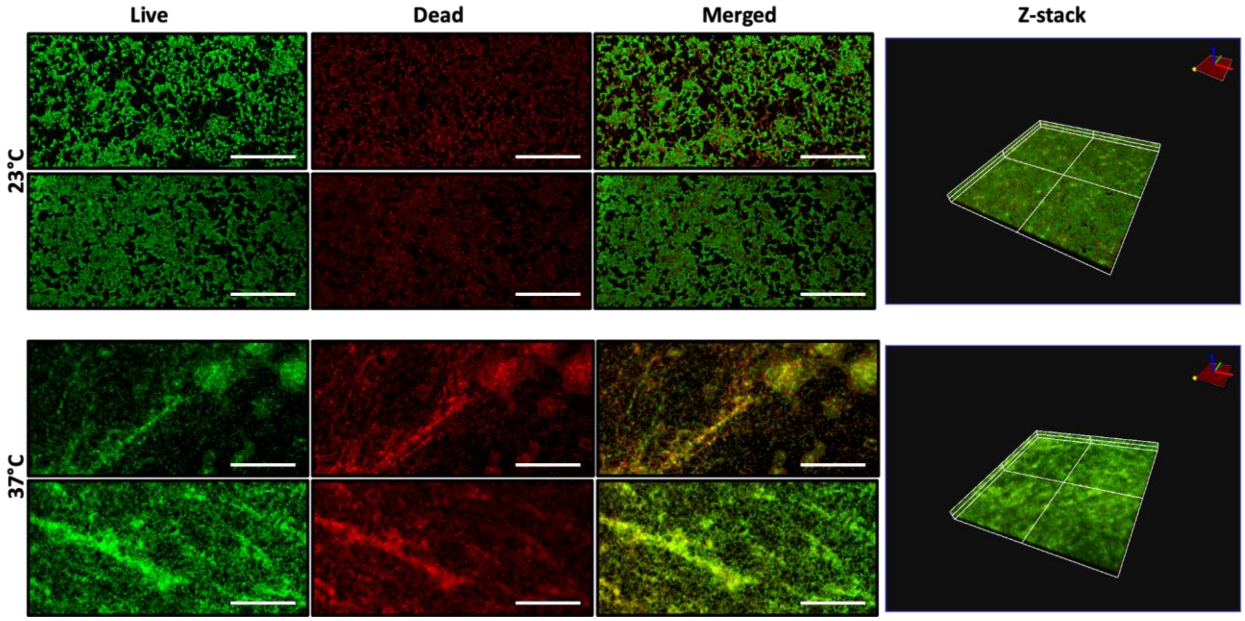

Supplementary Figure 1: The live/dead staining results of *P. aeruginosa* PA14 biofilm after 48 h of growth at 23°C and 37°C. Biofilm was grown for 48 h on a microscope glass slide at 23°C and 37°C and stained with FilmTracer LIVE/DEAD Biofilm Viability kit; SYTO 9 shows live cells in green and propidium iodide shows dead cells in red. Scale bar = 50 microns

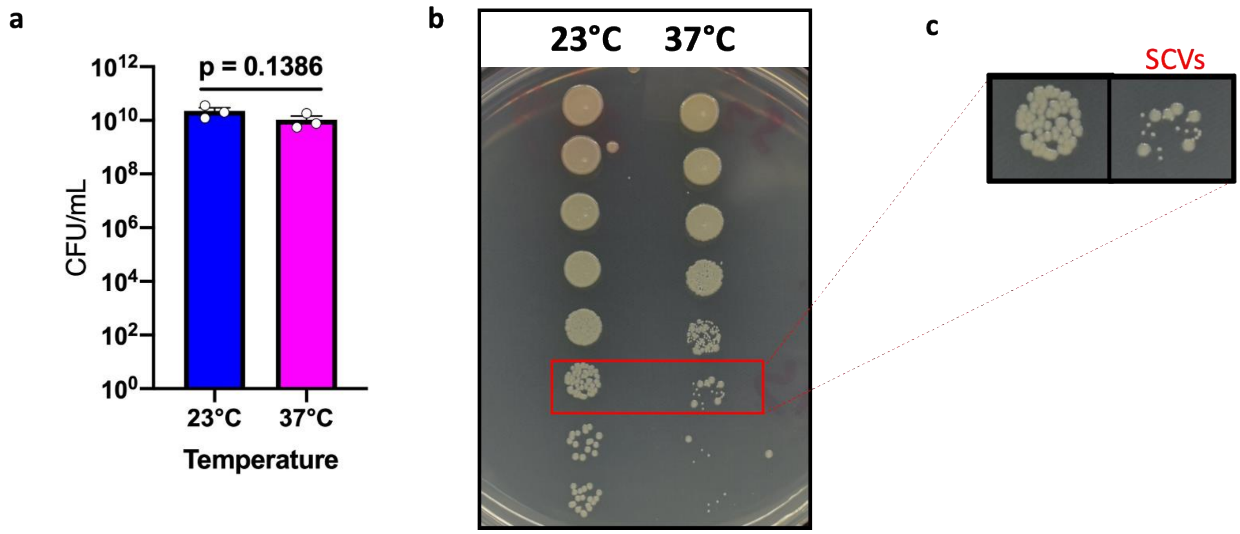

Supplementary Figure 2: *P. aeruginosa* biofilms were grown for 48 hours at both 23°C and 37°C after which planktonic cells were aspirated out of the tubes. 1X PBS was added to the tubes followed by sonication and plating the cells on Luria agar plates for cfu determination. **a.** A bar graph representing CFUs/mL for *P. aeruginosa* at both 23°C and 37°C. Bars represent the mean of three biological replicates performed on different days. The mean of each biological replicate was based on three technical replicates. Error bars represent the standard error of mean of the biological replicates. Unpaired t-test (two-tailed) was used to measure statistical significance. **b**. Representative serial dilution from which the data in the graph were derived. Small colony variants were observed as a subpopulation for *P. aeruginosa* cells grown at 37°C while only normal colony morphology was observed for cells grown at 23°C. **c**. Zoomed in image of the colonies at the two temperatures highlighting the presence of a subpopulation of small colony variants.

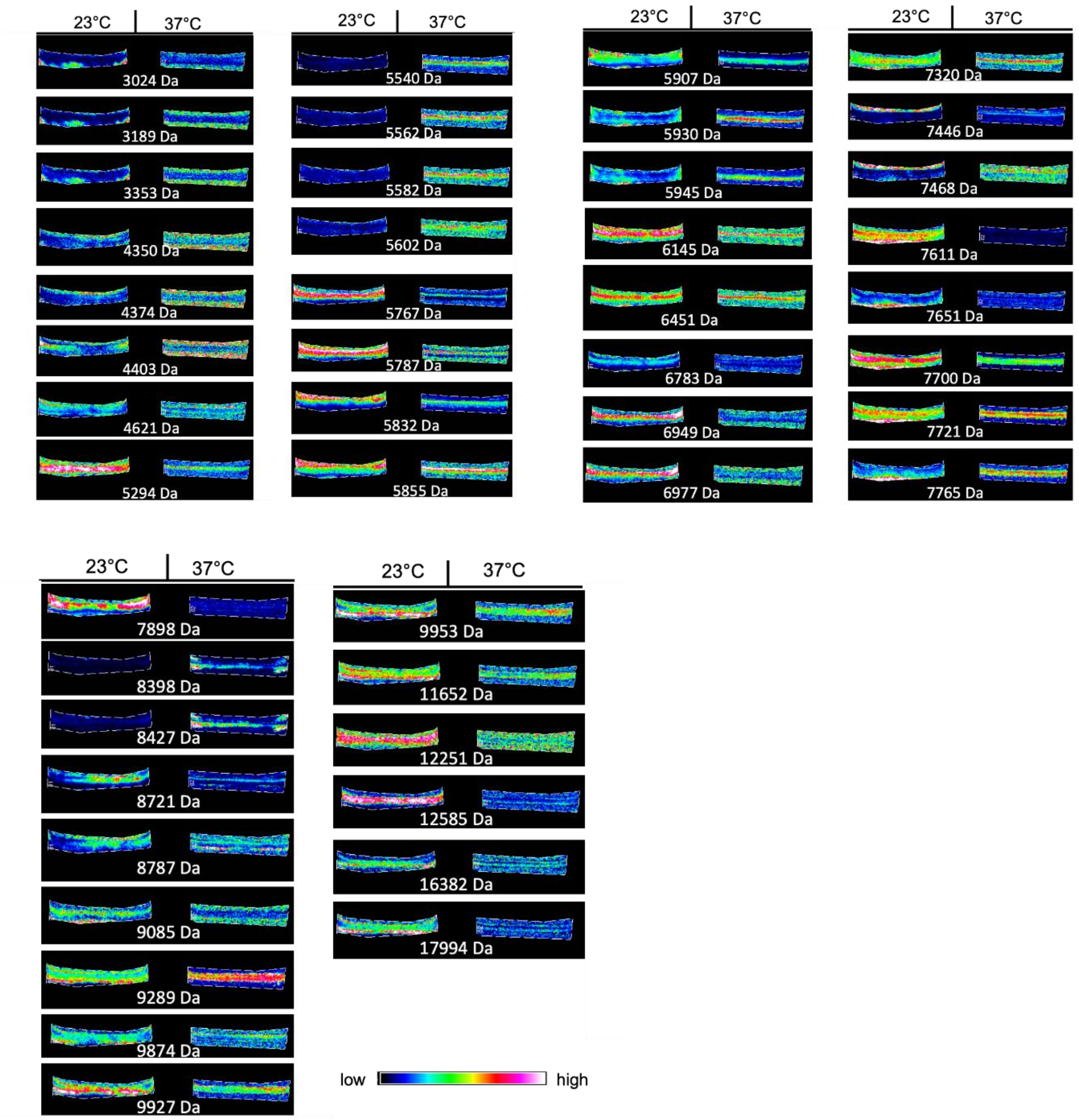

Supplementary Figure 3: Protein imaging using MALDI IMS showing the proteins enriched at host temperature and at environmental temperature in the biofilm formed by the pathogen *P. aeruginosa* UCBPP-PA14.

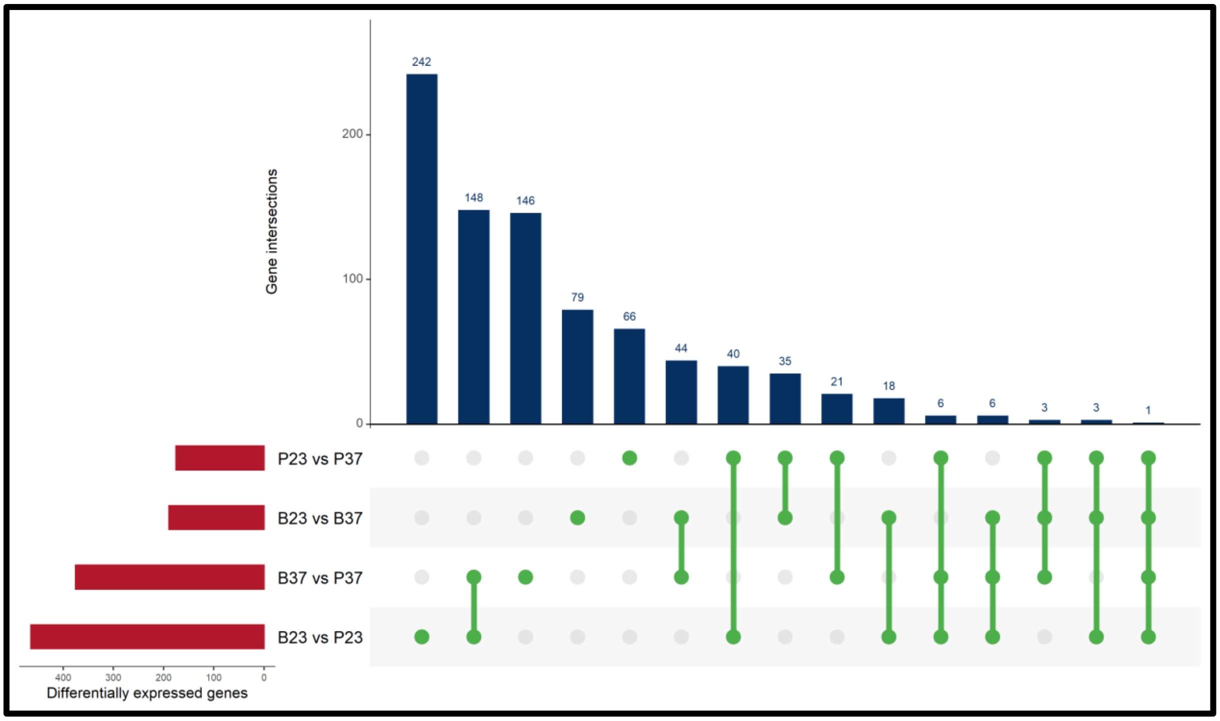

Supplementary Figure 4.

UpSet plot to summarize key differentially expressed (DE) genes across various categories. These panels summarize the DE gene overlap between common and exclusive differentially expressed genes (log2FC ≥ 2, q ≤ 0.05) between and amongst four growth and temperature categories of *P. aeruginosa*. The total number of genes differentially expressed for each category is shown in the bottom left horizontal red bar graph. The green color circles in each panel's matrix represent what would be the different Venn diagram sections, some of which are unique while other are overlapping DE genes). The green line connecting the circles indicate a certain intersection of DE genes between different categories. The top blue bar graph represents the number of differentially expressed genes for each unique or overlapping combination. P: planktonic, B: biofilm. (https://cran.rproject.org/web/packages/UpSetR/vignettes/basic.usage.html)

a

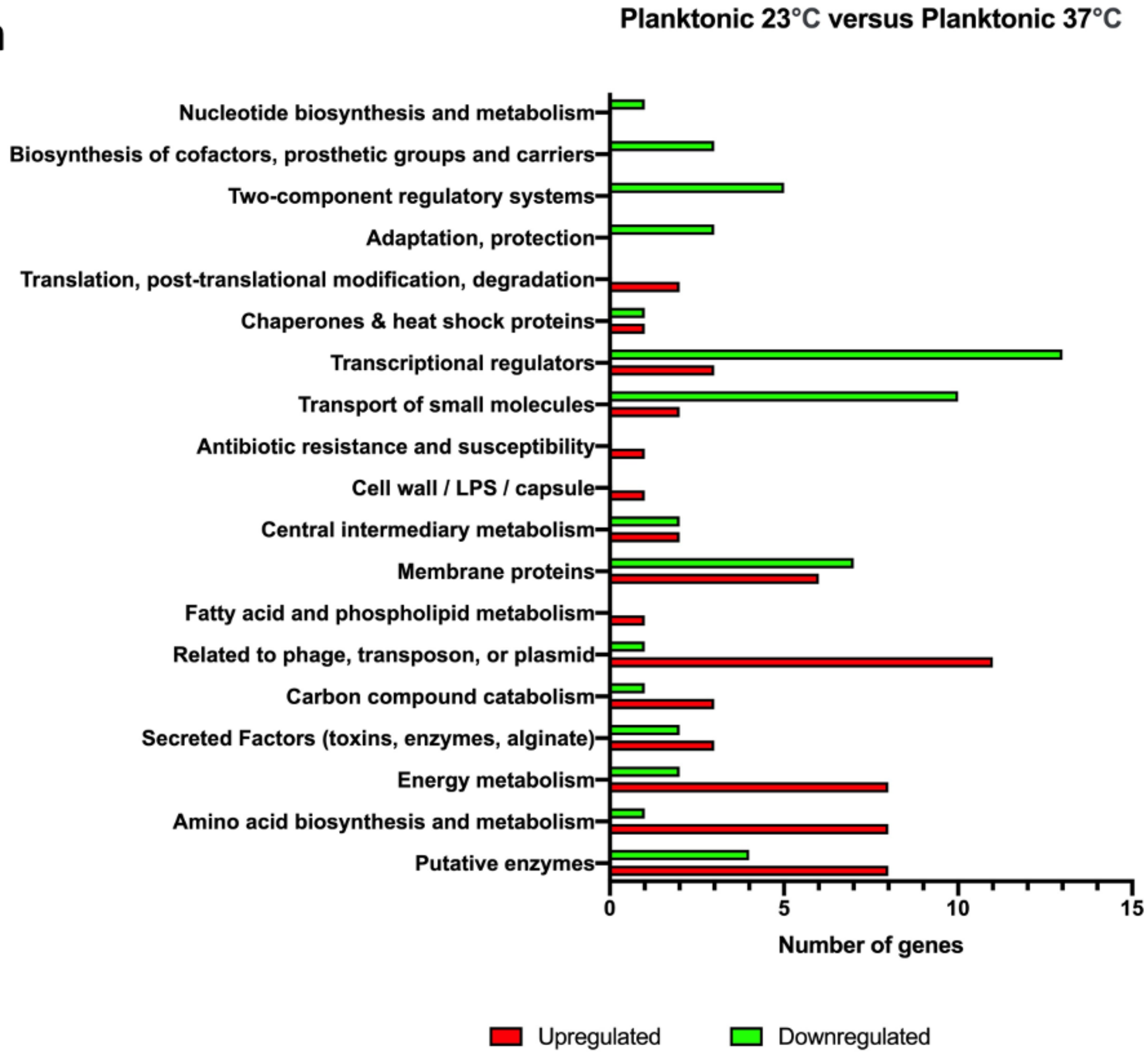

Planktonic 23°C versus Planktonic 37°C
Nucleotide biosynthesis and metabolism
Biosynthesis of cofactors, prosthetic groups and carriers
Two-component regulatory systems
Adaptation, protection
Translation, post-translational modification, degradation
Chaperones & heat shock proteins
Transcriptional regulators
Transport of small molecules
Antibiotic resistance and susceptibility
Cell wall / LPS / capsule
Central intermediary metabolism
Membrane proteins
Fatty acid and phospholipid metabolism
Related to phage, transposon, or plasmid
Carbon compound catabolism
Secreted Factors (toxins, enzymes, alginate)
Energy metabolism
Amino acid biosynthesis and metabolism
Putative enzymes
0
5
10
15
Number of genes
Upregulated
Downregulated

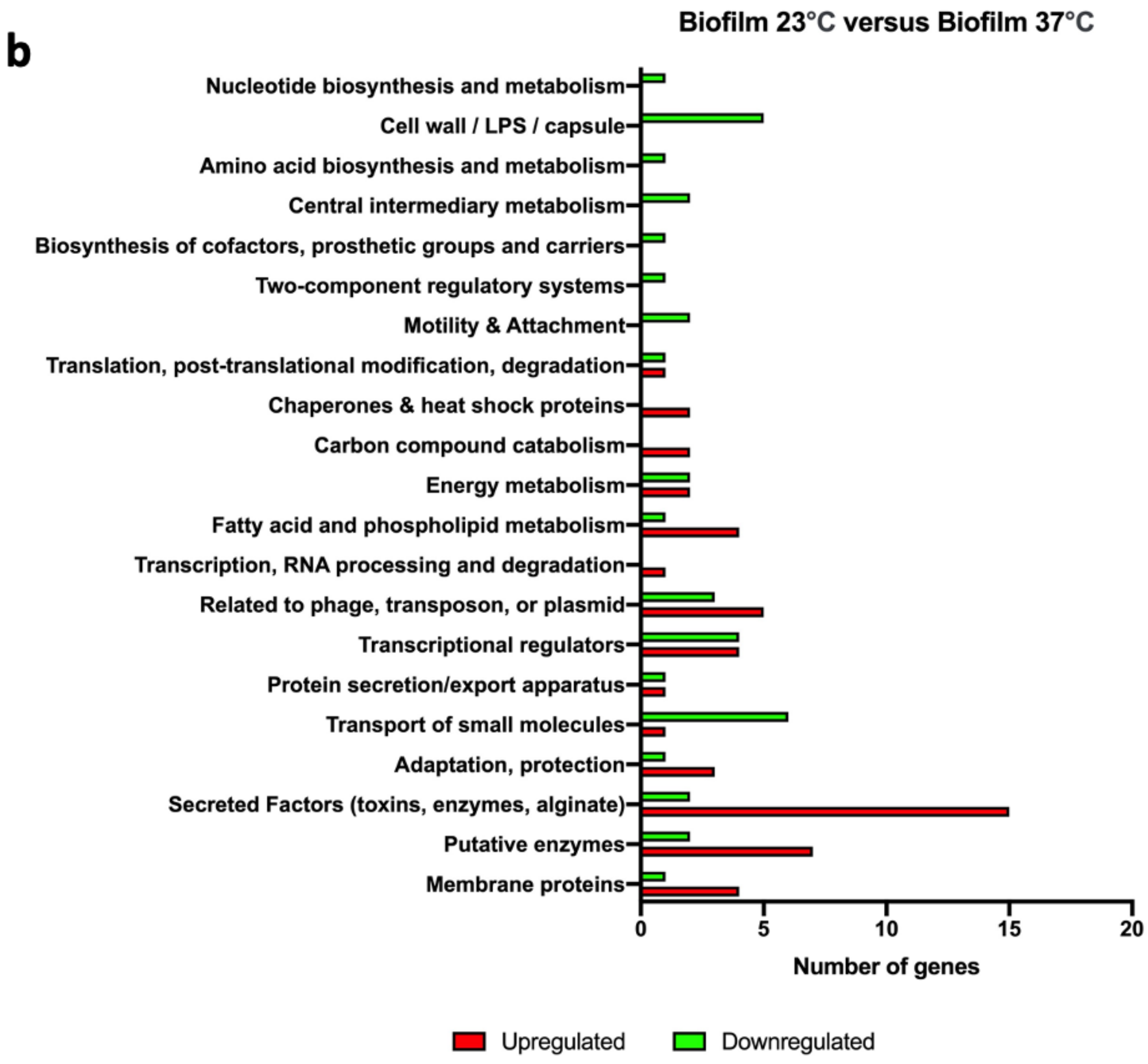

Supplementary Figure 5. **a**. Graph of differentially expressed genes and their regulation in the planktonic growth state at 23°C versus 37°C plotted using the PseudoCAP function class assignments. **b.** Graph of differentially expressed genes and their regulation in the biofilm growth state at 23°C versus 37°C plotted using the PseudoCAP function class assignments.

a

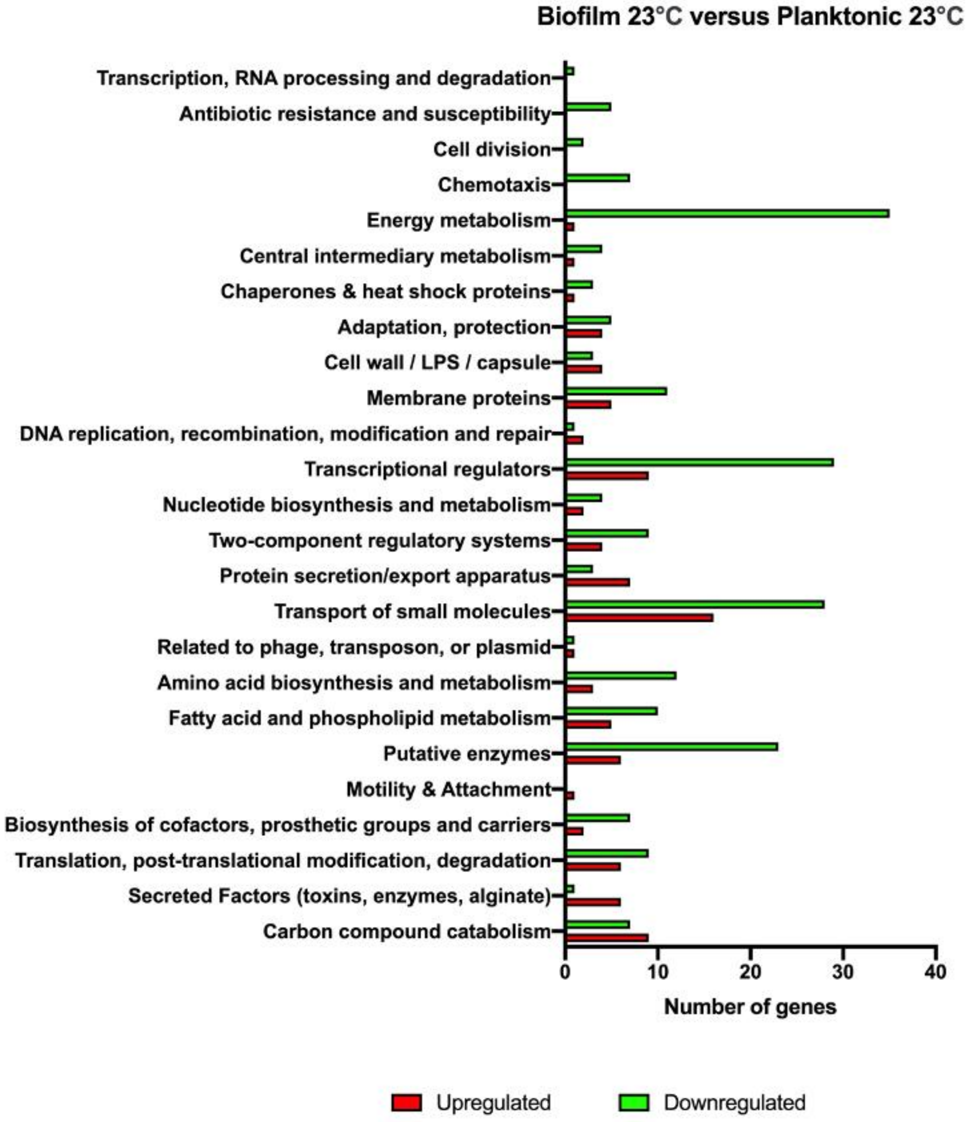
Biofilm 23°C versus Planktonic 23°C
Transcription, RNA processing and degradation
Antibiotic resistance and susceptibility
Cell division
Chemotaxis
Energy metabolism
Central intermediary metabolism
Chaperones & heat shock proteins
Adaptation, protection
Cell wall / LPS / capsule
Membrane proteins
DNA replication, recombination, modification and repair
Transcriptional regulators
Nucleotide biosynthesis and metabolism
Two-component regulatory systems
Protein secretion/export apparatus
Transport of small molecules
Related to phage, transposon, or plasmid
Amino acid biosynthesis and metabolism
Fatty acid and phospholipid metabolism
Putative enzymes
Motility & Attachment
Biosynthesis of cofactors, prosthetic groups and carriers
Translation, post-translational modification, degradation
Secreted Factors (toxins, enzymes, alginate)
Carbon compound catabolism
0
10
20
30
40
Number of genes
Upregulated
Downregulated

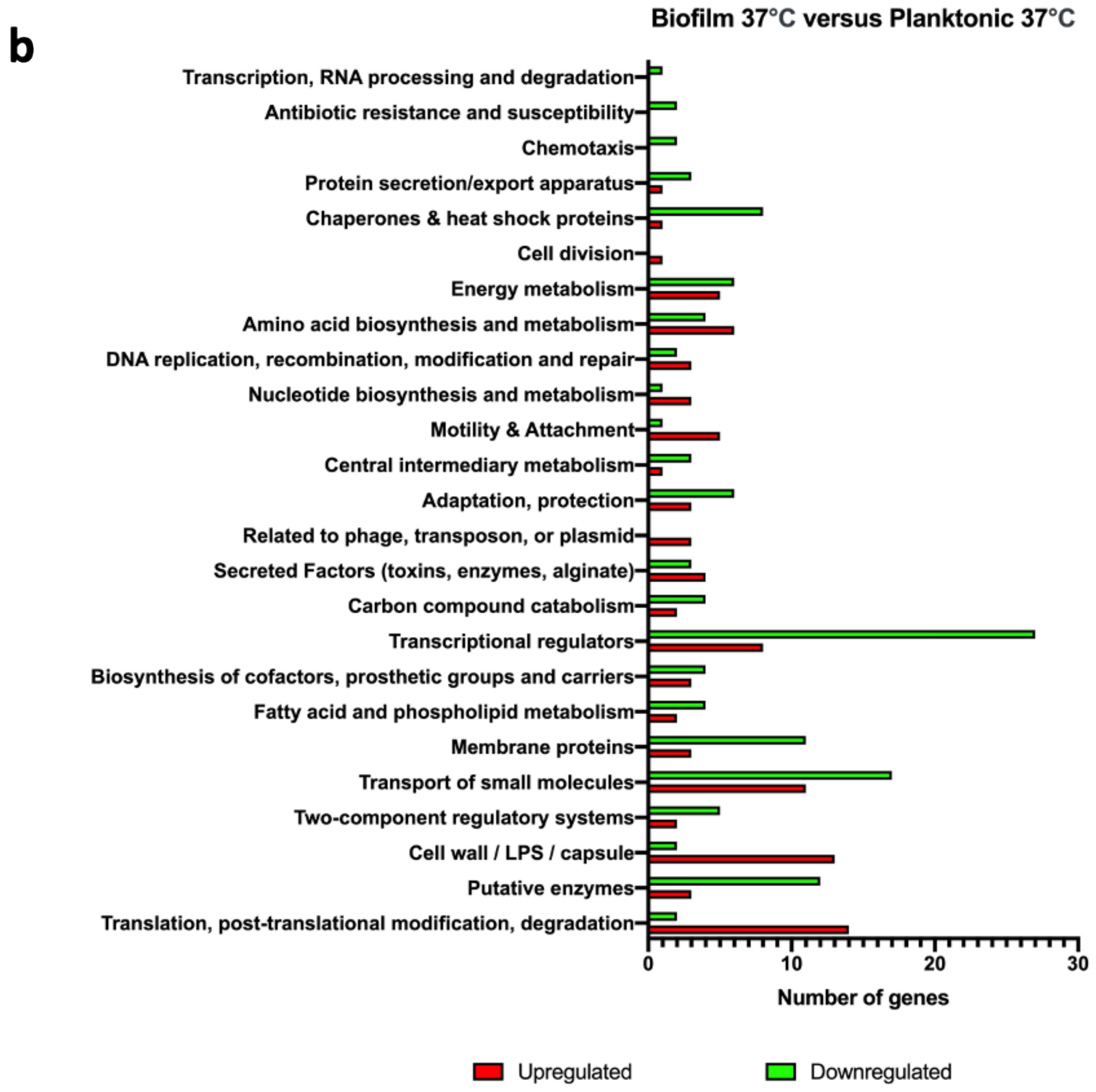

Supplementary Figure 6. **a**. Graph of differentially expressed genes and their regulation at 23°C for biofilm versus planktonic growth state plotted using the PseudoCAP function class assignments. **b.** Graph of differentially expressed genes and their regulation in the biofilm growth state at 37°C for biofilm versus planktonic growth state plotted using the PseudoCAP function class assignments.

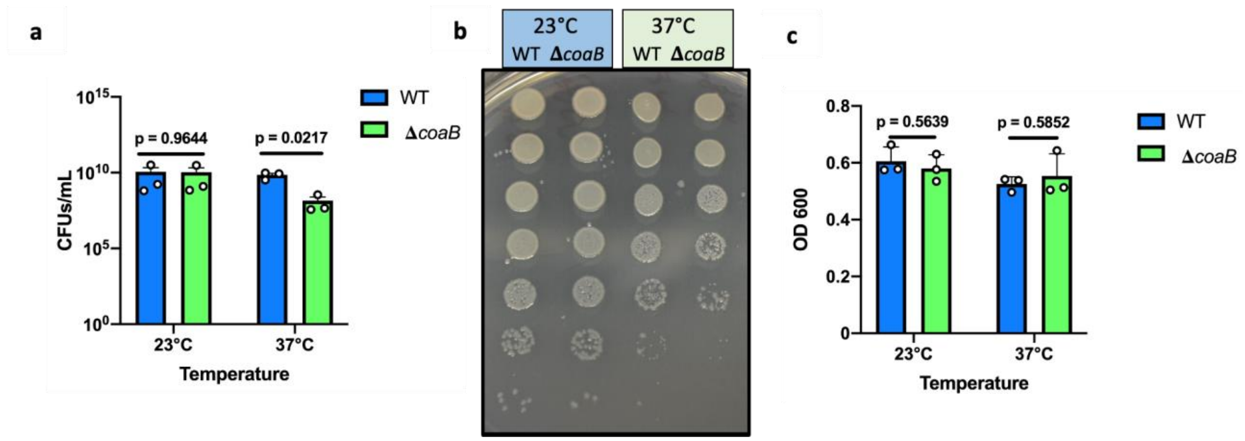

Supplementary Figure 7. a. Fewer viable cells were recovered from Δ*coaB* biofilms relative to WT biofilms grown at 37 °C, but the number of cells is unchanged in biofilms grown at 23 °C for both WT and Δ*coaB*. **b**. Small colony variants are still observed in Δ*coaB* biofilms at 37 °C at a rate approximately similar to WT PA14. **c**. While fewer viable cells were recovered from Δ*coaB* biofilms relative to WT biofilms grown at 37 °C, the overall cell numbers were equivalent at all conditions as determined by optical density readings at 600 nm. Bars represent the mean of three biological replicates performed on different days. The mean of each biological replicate was based on three technical replicates. Error bars represent the standard error of mean of the biological replicates. Unpaired t-test (two-tailed) was used to measure statistical significance.

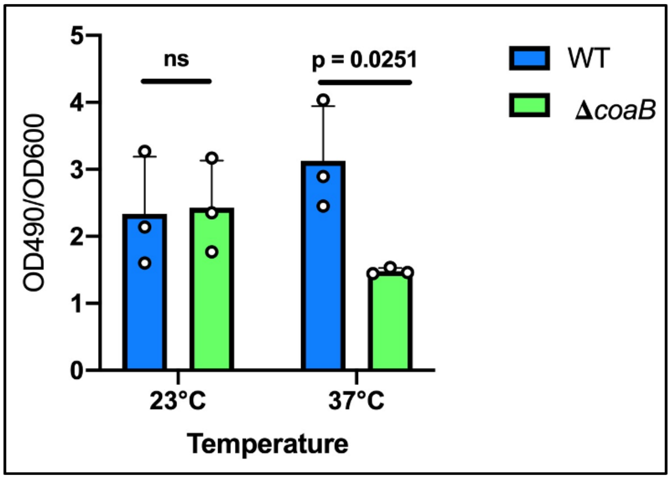

Supplementary Figure 8. Congo red binding assay. Extracellular matrix production by the wildtype and coaB mutant was quantified by add Congo Red (40 µg/mL) to the biofilm cells scraped off from microscope glass slides after 48 hours of growth at 23°C and 37°C. The data was normalized to OD 600 for each sample. Bars represent the mean of three biological replicates performed on different days. The mean of each biological replicate was based on three technical replicates. Error bars represent the standard error of mean of the biological replicates. Unpaired t-test (two-tailed) was used to measure statistical significance.

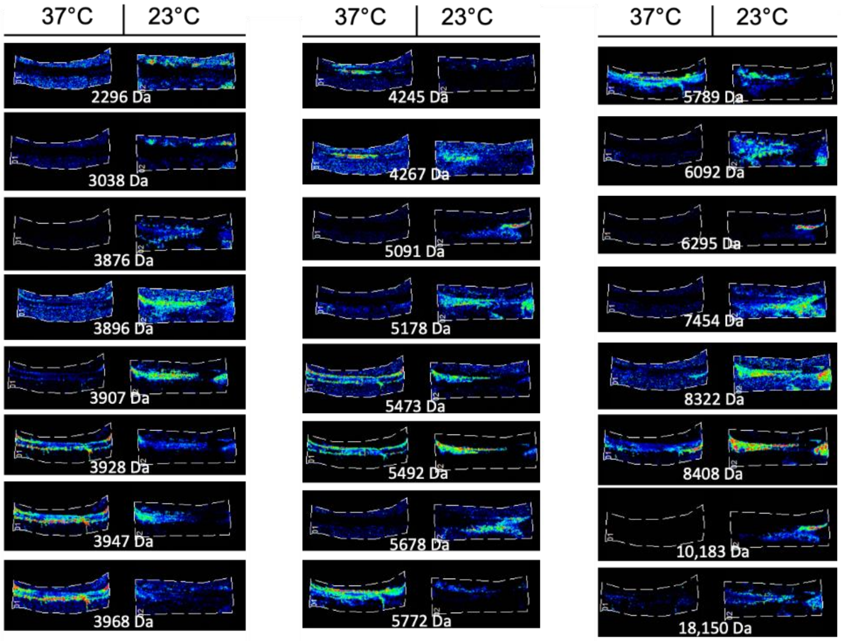

Supplementary Figure 9: Protein imaging using MALDI IMS showing the proteins enriched at host temperature and at environmental temperature in the biofilm formed by the pathogen *Acinetobacter baumanii* strain 17978.
