# Supplementary material for: Impact of temperature-dependent phage expression on Pseudomonas aeruginosa biofilm formation
Source: NPJ Biofilms Microbiomes. 2021 Mar 16;7:22. doi: 10.1038/s41522-021-00194-8 (PMC7966754; doi:10.1038/s41522-021-00194-8)
Supplement: Supplementary file 8 — Reporting Summary [file 41522_2021_194_MOESM8_ESM.pdf]

nature research

Corresponding author(s): Catherine A. Wakeman

Last updated by author(s): Dec 28, 2020

Reporting Summary

Nature Research wishes to improve the reproducibility of the work that we publish. This form provides structure for consistency and transparency in reporting. For further information on Nature Research policies, see our Editorial Policies and the Editorial Policy Checklist.

Statistics

For all statistical analyses, confirm that the following items are present in the figure legend, table legend, main text, or Methods section.

n/a	Confirmed	
☐	☒	The exact sample size (*n*) for each experimental group/condition, given as a discrete number and unit of measurement
☐	☒	A statement on whether measurements were taken from distinct samples or whether the same sample was measured repeatedly
☐	☒	The statistical test(s) used AND whether they are one- or two-sided<br>*Only common tests should be described solely by name; describe more complex techniques in the Methods section.*
☐	☒	A description of all covariates tested
☒	☐	A description of any assumptions or corrections, such as tests of normality and adjustment for multiple comparisons
☐	☒	A full description of the statistical parameters including central tendency (e.g. means) or other basic estimates (e.g. regression coefficient) AND variation (e.g. standard deviation) or associated estimates of uncertainty (e.g. confidence intervals)
☐	☒	For null hypothesis testing, the test statistic (e.g. *F*, *t*, *r*) with confidence intervals, effect sizes, degrees of freedom and *P* value noted<br>*Give P values as exact values whenever suitable.*
☒	☐	For Bayesian analysis, information on the choice of priors and Markov chain Monte Carlo settings
☒	☐	For hierarchical and complex designs, identification of the appropriate level for tests and full reporting of outcomes
☒	☐	Estimates of effect sizes (e.g. Cohen's *d*, Pearson's *r*), indicating how they were calculated

Our web collection on statistics for biologists contains articles on many of the points above.

Software and code

Policy information about availability of computer code

Data collection	FV Analysis software (Olympus FluoView 3000) (confocal imaging and z-stack); Hitachi PC-SEM software and Quartz PCI software (SEM micrograph); FlexImaging (MALDI IMS)
Data analysis	Rockhopper 2 (RNA-seq analysis); ImageJ (live/dead cell counting for confocal images); GraphPad Prism 8.0 (data analysis for CV, qPCR, and CFUS); FlexAnalysis (MALDI IMS)

For manuscripts utilizing custom algorithms or software that are central to the research but not yet described in published literature, software must be made available to editors and reviewers. We strongly encourage code deposition in a community repository (e.g. GitHub). See the Nature Research guidelines for submitting code & software for further information.

Data

Policy information about availability of data

All manuscripts must include a data availability statement. This statement should provide the following information, where applicable:

- Accession codes, unique identifiers, or web links for publicly available datasets
- A list of figures that have associated raw data
- A description of any restrictions on data availability

The raw RNA-seq data sets generated during this study are available through NCBI's BioProject database under accession number PRJNA664520. The raw data sets generated by MALDI IMS are available upon request. The authors declare that all other relevant data supporting the claims of the paper are available either in the main text or supplementary files.

Field-specific reporting

Please select the one below that is the best fit for your research. If you are not sure, read the appropriate sections before making your selection.

☒ Life sciences ☐ Behavioural & social sciences ☐ Ecological, evolutionary & environmental sciences

For a reference copy of the document with all sections, see nature.com/documents/nr-reporting-summary-flat.pdf

Life sciences study design

All studies must disclose on these points even when the disclosure is negative.

Sample size	All data sets were replicated on at least 3 independent days and performed in triplicate on each day. The same day triplicates were averaged and treated as technical replicates.
Data exclusions	No data exclusions were made.
Replication	All data sets were replicated on at least 3 independent days and performed in triplicate on each day. The same day triplicates were averaged and treated as technical replicates. By repeating experiments in triplicate both on the same day as well as performing these experiments on multiple days, we are confident in the reproducibility of these data.
Randomization	This is not relevant to our study.
Blinding	This was not performed as the same person collecting the data was performing the analysis for the bulk of the data sets. Therefore, blinding was not readily achievable.

Reporting for specific materials, systems and methods

We require information from authors about some types of materials, experimental systems and methods used in many studies. Here, indicate whether each material, system or method listed is relevant to your study. If you are not sure if a list item applies to your research, read the appropriate section before selecting a response.

Materials & experimental systems

n/a	Involved in the study	
☒	☐	Antibodies
☒	☐	Eukaryotic cell lines
☒	☐	Palaeontology and archaeology
☒	☐	Animals and other organisms
☒	☐	Human research participants
☒	☐	Clinical data
☒	☐	Dual use research of concern

Methods

n/a	Involved in the study	
☒	☐	ChIP-seq
☒	☐	Flow cytometry
☒	☐	MRI-based neuroimaging
